# Relationship between social determinants of health and processes and outcomes in adults with type 2 diabetes: validation of a conceptual framework

**DOI:** 10.1186/1472-6823-14-82

**Published:** 2014-10-09

**Authors:** Rebekah J Walker, Mulugeta Gebregziabher, Bonnie Martin-Harris, Leonard E Egede

**Affiliations:** Health Equity and Rural Outreach Innovation Center (HEROIC), Charleston VA HSR&D COIN, Ralph H. Johnson VAMC, Charleston, SC USA; Center for Health Disparities Research, Medical University of South Carolina, 135 Rutledge Avenue, Room 280, PO Box 250593, Charleston, SC USA; Department of Health Science and Research, Medical University of South Carolina, Charleston, SC USA; Division of Public Health Sciences, Department of Medicine, Medical University of South Carolina, Charleston, SC USA; Department of Otolaryngology-Head and Neck Surgery, Medical University of South Carolina, Charleston, SC USA; Division of General Internal Medicine and Geriatrics, Department of Medicine, Medical University of South Carolina, Charleston, SC USA

**Keywords:** Diabetes, Social determinants, Socioeconomic, Psychological, Glycemic control, Conceptual framework

## Abstract

**Background:**

The aim of this study was to empirically validate a conceptual framework and elucidate the pathways linking social determinants of health to outcomes in individuals with type 2 diabetes.

**Methods:**

615 adults were recruited from adult primary care clinics in the southeastern United States. The model was estimated using path analysis to determine if socioeconomic (education, employment, income) and psychosocial (fatalism, self-efficacy, depression, diabetes distress, serious psychological distress, social support, and perceived stress) factors would independently predict glycemic control or be associated with mediator/moderators of self-care, access to care, and processes of care. Covariates were gender, age, race and health literacy.

**Results:**

The final model (chi2 (15) = 17.68, p = 0.28; RMSEA = 0.02, CFI = 0.99) showed lower glycemic control was directly associated with less hours worked (r = 0.13, p = 0.002), more fatalistic attitudes (r = −0.09, p = 0.03), more self-efficacy (r = −0.30, p < 0.001), and less diabetes distress (r = 0.12, p = 0.03), with the majority of total effects being direct. Significant paths associated self-care with diabetes distress (r = −0.14, p = 0.01) and perceived stress (r = −0.15, p = .001); access to care with income (r = 0.08, p = 0.03), diabetes distress (r = −0.21, p < 0.001) and social support (r = 0.08, p = 0.03); and processes of care with income (r = −0.11, p = 0.03), social support (r = 0.10, p = 0.04), and perceived stress (r = 0.10, p = 0.04). The paths explained 76% of the variance in the model.

**Conclusions:**

Consistent with the conceptual framework, social determinants were associated with glycemic control through a direct association and mediators/moderators of self-care, access to care and processes of care. This study provides the first validation of a conceptual framework for the relationship between socioeconomic and psychological components of social determinants of health and diabetes outcomes.

## Background

Worldwide, diabetes has caused 5.1 million deaths, while treatment of the 382 million people living with diabetes cost 548 billion dollars in healthcare spending [1]. In the United States alone, 29.1 million people, or 9.3% of the population have diabetes, and healthcare spending in 2012 as a result of diabetes totaled 245 billion dollars [2]. While it is the seventh leading cause of death in the United States, nearly 80% of those diagnosed with diabetes live in low and middle-income countries [1, 2]. Studies have shown that poor glycemic control is associated with adverse outcomes, including reduction in risk of micro vascular complications and cardiovascular disease [3]. Most interventions focus on lifestyle changes in order to improve glycemic control, which though effective at an individual level remains a challenge at the population level. Based on a recent analysis of trends in diabetes in the United States, while use of oral medications increased and glycemic control improved overall, a substantial portion of the United States population does not have optimal glycemic control [4]. Further efforts are needed to address the barriers limiting patients with type 2 diabetes.

A lack of attention to the importance of social determinants of health, or the social and economic conditions that influence health, has been suggested as a reason for the lack of population level change in diabetes outcomes [5–8]. Social determinants of health have been associated with increased incidence, prevalence and burden of disease, and impact the health and well-being of individuals and populations [9–11]. Understanding the causal pathway of social determinants of health is now recognized as a critical aspect for understanding the root cause for health problems and developing effective health interventions [12–14]. A review by the World Health Organization (WHO) investigating pathways between social conditions and health outcomes found that perceptions and experiences of individuals, including material factors, psychosocial factors, behavioral/biological factors and the health system, may influence health [11].

In 2004, Brown et al. developed a conceptual framework for the mechanisms connecting socioeconomic factors and health in individuals with diabetes [15]. This framework incorporates individual, household and neighborhood socioeconomic status as a predictor of both general and diabetes-specific outcomes. While socioeconomic status is an important predictor of diabetes outcomes [16], additional social determinants such as psychosocial influences are important to consider when investigating pathways [17–19]. With minor adaptation, the Brown et al. model can provide a way to elucidate the pathways linking social determinants of health factors to health outcomes in individuals with type 2 diabetes.

The aim of this study was to validate a modified version of the Brown et al. [15] model to explain the relationship between socioeconomic and psychosocial components of social determinants of health and diabetes outcomes. We hypothesized based on the model that socioeconomic and psychological components of social determinants of health will be significantly associated with glycemic control both directly and indirectly through self-care, access to care, and processes of care.

## Methods

### Sample

**Figure 1:**
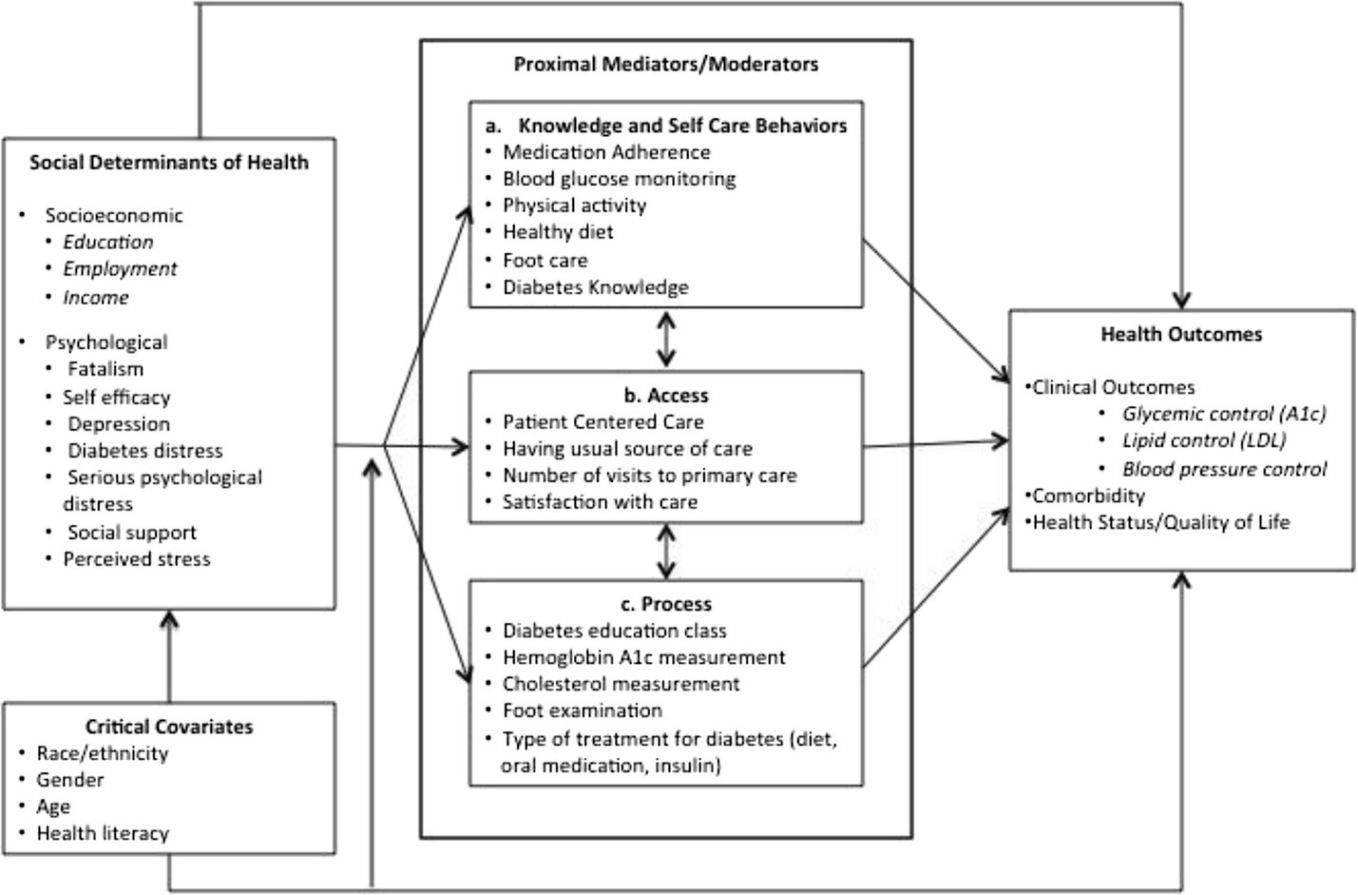
**Modified model adapted from Brown et al. (2004)** [15] **for the relationship between socioeconomic and psychosocial social determinants of health factors and health outcomes in patients with type 2 diabetes.**

Ethics approval was obtained through our local Institutional Review Board (Medical University of South Carolina Office of Research Integrity) and informed consent was obtained from all participants. 615 adults diagnosed with type 2 diabetes were recruited from two adult primary care clinics in the southeastern United States. Eligibility included ages 18 years or older, diagnosis of type 2 diabetes in their medical record, and ability to communicate in English. Patients were ineligible if through interaction or chart documentation patients were determined to be cognitively impaired as a result of significant dementia or active psychosis. Letters of invitation were sent in addition to approaching patients in the clinic waiting rooms. Research coordinators provided a detailed explanation of the study and consented patients. Participants then completed validated questionnaires that captured social determinants of health factors, demographic information and self-care information. Validated questionnaires were included based on the modified version of the conceptual framework by Brown et al. (Figure 1). HbA1c was abstracted from the electronic medical record as an outcome measure.

### Social determinants of health variables

#### Socioeconomic status

Previously validated items from the 2002 National Health Interview Survey [20] were used to capture household income, years of education and employment status. Household income was categorized into 4 income units: <$20,000, $20,000-$49,999, $50,000-$74,999, ≥ $75,000. Years of education were categorized into 4 units: less than high school, high school graduate, college education, and more than college education. Employment was dichotomized as not employed and employed.

#### Fatalism

Fatalism was assessed with the Diabetes Fatalism Scales (DFS); a 12-item scale where higher scores represent greater diabetes fatalism [21]. The DFS has a Cronbach’s alpha of 0.80 [21].

#### Self-efficacy

Self-efficacy was assessed with the Perceived Diabetes Self-Management Scale (PDSMS); an 8-item measure where higher scores indicate higher self-efficacy [22]. It is a valid and reliable measure of diabetes self efficacy (Cronbach alpha = 0.83).

#### Depression

Depression was assessed with the PHQ-9; a 9-item scale based on the DSM-IV criteria for depression with higher scores indicating higher depression [23]. Sensitivity is 88% and specificity is 88% for major depression [24].

#### Diabetes distress

Distress was assessed with the Diabetes Distress Scale (DDS); a 17-item measure with questions about disease management, support, emotional burden and access to care [25]. The sensitivity and specificity ranged from 0.85 to 0.97 [25].

#### Serious psychological distress

Serious Psychological Distress (SPD) was assessed with the K6; a 6-item scale with higher scores representing higher probability of severe mental illness. The scale has good precision and consistent psychometric properties across major socio-demographic samples [26].

#### Social support

Social Support was assessed with the Medical Outcomes Study (MOS) Social Support Survey; a 19-item scale measuring tangible support, affection, positive social interaction, and emotional or informational support. The total scale has high internal consistency (α = 0.97), good criterion and discriminant validity, and one-year test-retest reliability (0.72 to 0.76) [27].

#### Perceived Stress

Stress was assessed with the Perceived Stress Scale (PSS); a 4-item scale assessing the frequency over the previous month with which the respondent finds situations stressful [28]. The Cronbach alpha value is 0.69 and scores are highly correlated with stress, depression and anxiety [29].

### Diabetes processes and outcomes

#### Diabetes knowledge

Diabetes Knowledge was assessed with the Diabetes Knowledge Questionnaire (DKQ); a 24-item scale where the final score is based on the percentage of correct scores [30].

#### Self-reported medication adherence

Medication Adherence was assessed with the Morisky Medication Adherence Scale (MMAS); an 8-item scale with higher values indicating higher adherence [31].

#### Behavioral skills

Diabetes behavior was assessed with the Summary of Diabetes Self-Care Activities (SDSCA) scale; an 11-item scale measuring frequency of self-care activity in the last 7 days for general diet (follow healthy diet), specific diet (ate fruits/two fat diet), exercise, blood glucose testing, and foot care [32].

#### Medical access

Previously validated items from the 2010 Medical Expenditure Panel Survey – Household Component [33] and Behavioral Risk Factor Surveillance System [34] were used to capture having a usual source of care, the number of visits to primary care, patient-centered care, and satisfaction with care.

#### Medical process

Previously validated items from the 2010 Medical Expenditure Panel Survey – Household Component [33] were used to capture frequency of measurement of HbA1c and cholesterol, examinations of feet by a provider, type of treatment used for diabetes, and attendance in diabetes education classes.

#### Clinical measures

Hemoglobin A1c was abstracted from the electronic medical record using values within the previous 6 months.

### Statistical analyses

Descriptive statistics were performed to describe the data and ensure data were multivariate normal, linearly related and at least interval scale to meet assumptions [35]. The hypothesized model was then estimated using path analysis in STATA version 13, which allows structural equation modeling using the maximum likelihood estimation procedure. The ‘mlmv option’ in STATA version13 was used, which retains variables rather than using listwise deletion. Retaining variables, and a sample size of 600 adults provides the recommended 20:1 ratio (subjects to variables) necessary to maintain 80% power while estimating parameters and standard errors [36, 37]. This sample size minimizes the likelihood of over-saturating the model, while providing stability of parameter estimates [36, 38].

The modified Brown et al. model was used to conceptualize the hypothesis that socioeconomic (education, employment, income) and psychosocial (fatalism, self-efficacy, depression, diabetes distress, serious psychological distress, social support, and perceived stress) factors would be independent predictors of self-care (medication adherence, general diet, specific diet, exercise, blood sugar testing, foot care), access to care (health care access, visits to primary care, patient centered care, satisfaction with care), processes of care (A1c testing, cholesterol testing, foot checks by a physician, treatment for diabetes, diabetes education classes) and glycemic control. In addition, self-care, access to care and processes of care would be independent predictors of glycemic control. Gender, age, race and health literacy level were included as covariates. All analyses were completed using standardized estimates, which are interpreted as the change in standard deviation of the outcome due to one standard deviation increase in the predictor, and are useful when variable scales are dissimilar as is the case in this study [39].

Hypotheses regarding specific structural relationships between constructs in the model were evaluated through inspection of direction and magnitude of path coefficients. Path analysis permits the inclusion of multiple independent and multiple dependent variables, providing flexibility for simultaneous regression models, and insight into direct and indirect effects of variables [36]. A model is based on a priori specifications based on hypotheses and analyzed to determine if the model is supported by the data, or if alternative models can exist [40]. Pathways between all variables in the model were included to verify factors with no association. Model fit was evaluated using the chi2 statistic, root mean square error of approximation (RSMEA) and comparative fit index (CFI). A model with a non-significant chi2 statistic indicates good fit. RSMEA ranges from 0 to infinity, with values lower than 0.05 indicating good fit, and 0.07 indicating reasonable fit [41]. CFI ranges from 0 to 1, where 1 indicates perfect data fit, 0.95 indicates good fit, and 0.90 indicates adequate fit [41].

## Results

### Sample demographics

Demographic characteristics for this sample of 615 adults with type 2 diabetes are shown in Table 1. The mean age was 61 years, with the majority being men (61.6%), non-Hispanic black (64.9%), and employed (65.3%). 13% had less than a high school diploma, and 41.6% earned less than $20,000 annually. Descriptive information on self-care and psychological measures included in the model are also presented in Table 1.

**Table 1:** **Sample demographic characteristics and path analysis variables (n = 615)**

	% or mean ± standard deviation
Age	61.3 ± 10.9
18-34 years	1.6
35-44 years	5.2
45-64 years	53.6
65+ years	39.6
Gender	
Women	38.4
Men	61.6
Race/Ethnicity	
Non-Hispanic Black	64.9
Non-Hispanic Whites	33.0
Hispanic/Other	2.1
Marital Status	
Married	49.7
Not Married	50.3
Educational level	
Less than high school graduate	13.0
High school graduate	28.2
College education	47.1
More than college	11.7
Employment status	
Employed	34.7
Not employed	65.3
Annual income level	
<$20,000	41.6
$20,000-$49,000	10.1
$50,000-$74,999	9.4
$75,000+	7.9 ± 1.8
Glycemic Control	
Glycemic Control (HbA1c < 8%)	
Controlled	57.9
Not Controlled	42.1
Self-Care	
General Diet	4.7 ± 2.0
Special Diet	4.0 ± 1.5
Exercise	2.6 ± 2.2
Blood Sugar Testing	4.6 ± 2.5
Foot Care	4.3 ± 2.5
Medication Adherence	5.9 ± 2.0
Psychological Factors	
Fatalism	33.9 ± 9.5
Self-efficacy	28.6 ± 5.4
Depression	6.1 ± 6.0
Diabetes Distress	1.6 ± 0.7
Serious Psychological Distress	5.8 ± 6.3
Social Support	72.8 ± 26.1
Perceived Stress	5.3 ± 3.3

### Validation of the conceptual framework

**Figure 2:**
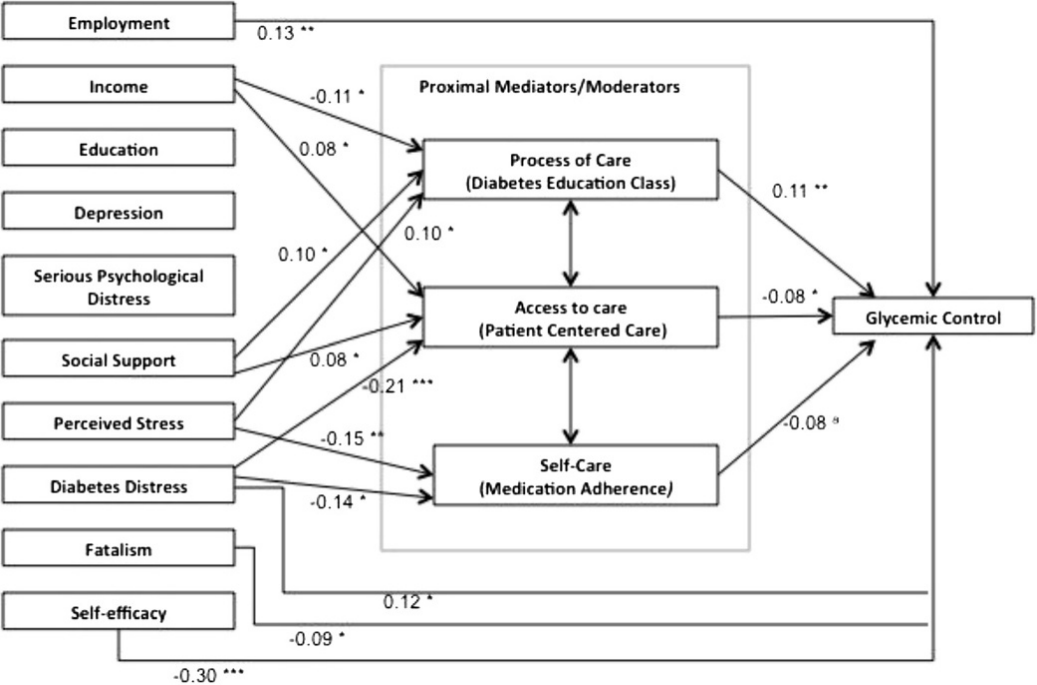
**Path model of social determinants of health on glycemic control, adjusting for age, gender, race and health literacy.** Overall model fit chi2 (15) = 17.68, p = 0.28; RMSEA = 0.02 (90% CI 0.00, 0.04), CFI = 0.99. ^a^p = 0.06, *p < 0.05, **p < 0.01, ***p < 0.001. Note: coefficient for path between access to care and glycemic control is based on visits to primary care rather than patient centered care.

The estimated model demonstrated good data fit, chi2 (15) = 17.68, p = 0.28; RMSEA = 0.02 (90% CI 0.00, 0.04) and CFI = 0.99. Standardized direct, indirect and total effects of the path analysis are shown in Tables 2 and 3. While all measures hypothesized as being part of the model in Figure 1 were included in the analysis, for the sake of parsimony and interpretation indicator variables for each mediator/moderator were chosen as shown in Figure 2. In addition, non-significant paths were retained in the model rather than presenting results of a trimmed model to provide information on the full conceptual framework.

**Table 2:** **Standardized effects of social determinants of health, self-care, access to care and processes of care on glycemic control, controlling for age, gender, race and health literacy**

	Direct effect	Indirect effect	Total effect
**Glycemic control**			
➔ Social Determinants			
Education	−0.01	0.001	−0.01
Employment	0.12**	0.01	0.13**
Income	−0.06	−0.008	−0.07
Fatalism	−0.08	−0.01	−0.09*
Self-efficacy	−0.29***	−0.01	−0.30***
Depression	−0.04	−0.005	−0.04
Diabetes distress	0.11*	0.01	0.12*
SPD	0.03	0.003	0.04
Social support	0.05	0.02*	0.07
Perceived stress	−0.05	0.02	−0.03
➔ Self-care			
Medication adherence	−0.08^a^	-	−0.08^a^
Knowledge	0.03	−0.002	0.03
General diet	0.05	−0.004	0.05
Special diet	0.03	−0.01	0.02
Exercise	−0.06	−0.003	−0.06
Blood sugar testing	0.01	−0.005	0.002
Foot care	0.01	−0.01	0.001
➔ Access to care			
Patient centered care	0.05	−0.003	0.04
Usual source of care	−0.04	−0.003	−0.04
Visits to primary care	−0.08*	−0.004	−0.08*
Satisfaction with care	−0.03	0.02	−0.01
➔ Processes of care			
Diabetes education	0.10**	0.006	0.11**
A1c in 12 mo.	−0.003	0.002	−0.002
Cholesterol in 12 mo.	0.04	0.01	0.05
Foot check in 12 mo.	0.05	−0.0001	0.05
Treatment with diet	−0.01	0.01*	0.0002
Treatment with oral	0.02	0.01*	0.03
Treatment with insulin	0.26***	0.03*	0.28***

Note: the significance levels shown here are for the unstandardized solution.

^a^p = 0.06; *p < 0.05; **p < 0.01; and ***p < 0.001.

**Table 3:** **Standardized effects of social determinants of health on mediator/moderators of self-care, access to care and processes of care, controlling for age, gender, race and health literacy**

	Direct effect	Indirect effect	Total effect
**Self-care**			
➔ Social Determinants			
Education	−0.07	0.005	−0.07
Employment	−0.05	−0.005	−0.05
Income	−0.01	0.01	0.003
Fatalism	0.07	0.001	0.07
Self-efficacy	0.06	0.003	0.06
Depression	−0.08	0.01	−0.07
Diabetes distress	−0.13*	−0.01	−0.14**
SPD	0.01	−0.003	0.003
Social support	−0.07	−0.005	−0.07
Perceived stress	−0.14**	−0.01	−0.15**
➔ Access to care			
Patient centered care	0.04	-	0.04
Usual source of care	0.06	0.002	−0.04*
Visits to primary care	0.06	0.001	−0.08
Satisfaction with care	0.05	0.02	−0.01
➔ Processes of care			
Diabetes education	−0.08*	−0.0002	0.11**
A1c in 12 mo.	0.005	−0.002	−0.002
Cholesterol in 12 mo.	0.002	−0.005	0.05
Foot check in 12 mo.	0.06	−0.001	0.05
Treatment with diet	−0.04	−0.003	0.0002
Treatment with oral	−0.08*	−0.006	0.03
Treatment with insulin	−0.03	−0.02*	0.28***
**Access to care**			
➔ Social Determinants			
Education	0.01	0.0002	0.02
Employment	−0.005	−0.0003	−0.01
Income	0.08*	0.0005	0.08*
Fatalism	−0.04	0.0002	−0.04
Self-efficacy	−0.01	0.0002	−0.01
Depression	0.03	0.0005	0.03
Diabetes distress	−0.21***	−0.0001	−0.21***
SPD	−0.002	−0.0001	−0.002
Social support	0.08*	−0.0004	0.08*
Perceived stress	−0.06	−0.0004	−0.06
➔ Processes of care			
Diabetes education	−0.004	-	−0.004
A1c in 12 mo.	−0.01	−0.0001	−0.009
Cholesterol in 12 mo.	0.03	−0.0003	0.03
Foot check in 12 mo.	0.03	−0.0001	0.03
Treatment with diet	0.09**	−0.0003	0.08**
Treatment with oral	−0.01	−0.0003	−0.01
Treatment with insulin	−0.02	−0.001	−0.02
**Processes of care**			
➔ Social Determinants			
Education	−0.05	-	−0.05
Employment	0.06	-	0.06
Income	−0.11*	-	−0.11*
Fatalism	−0.04	-	−0.04
Self-efficacy	−0.04	-	−0.04
Depression	−0.11	-	−0.12
Diabetes distress	0.03	-	0.03
SPD	0.03	-	0.03
Social support	0.10*	-	0.10*
Perceived stress	0.10*	-	0.10*

Note: the significance levels shown here are for the unstandardized solution. *p < 0.05; **p < 0.01; ***p < 0.001.

As indicated in Table 2 and Figure 2, there were significant total effects of socioeconomic and psychosocial factors on glycemic control for employment (r = 0.13, p = 0.002), fatalism (r = −0.09, p = 0.03), self-efficacy(r = −0.30, p < 0.001), and diabetes distress (r = 0.12, p = 0.03), such that less hours worked, more fatalistic attitudes, more self-efficacy, and less diabetes distress were associated with lower HbA1c. The majority of the total effects were direct: 92% for employment, 88% for fatalism, 93% for self-efficacy, and 92% for diabetes distress.

While the direct and total effects for social support were not significant, the indirect effect was (r = 0.02, p = 0.03). Table 3 shows that this indirect effect is mediated by access to care and process of care, where there was a significant total effect of social support on access to care (r = 0.08, p = 0.03) and processes of care (r = 0.01, p = 0.04). 100% of these effects are direct.

As indicated in Table 3 and Figure 2, there were also significant total effects of socioeconomic and psychosocial factors on self-care (medication adherence) for diabetes distress (r = −0.14, p = 0.01) and perceived stress (r = −0.15, p = 0.001), such that lower diabetes distress and perceived stress is associated with higher self-care. There were significant total effects of socioeconomic and psychosocial factors on access to care (patient centered care) for income (r = 0.08, p = 0.03), diabetes distress (r = −0.21, p < 0.001) and social support (r = 0.08, p = 0.03), such that higher income, lower diabetes distress, and higher social support were associated with higher access. Lastly, there significant total effects of socioeconomic and psychosocial factors on processes of care (diabetes education in past 12 months) for income (r = −0.11, p = 0.03), social support (r = 0.10, p = 0.04), and perceived stress (r = 0.10, p = 0.04) such that lower income, higher social support and higher perceived stress were associated with higher processes of care. The majority of all these paths were direct effects (93-100% for each).

Overall, the model explained 29% of the variance in HbA1c, 30% of the variance in self-care (medication adherence), 53% of the variance in access to care (patient centered care), 16% of processes of care (diabetes education in the past 12 months), and 76% of the variance overall.

## Discussion

Consistent with the conceptual framework, socioeconomic and psychological social determinants of health were associated with glycemic control through a direct association and through the mediators/moderators of self-care, access to care and processes of care. The socioeconomic variables employment and income showed significant associations, such that less hours worked were associated with a lower HbA1c and higher income was associated with higher access and lower processes of care. Higher fatalism, higher self-efficacy and lower diabetes distress were directly associated with lower HbA1c, and social support showed a significant indirect effect on glycemic control mediated by access to care and processes of care. Lower diabetes distress, higher social support and lower perceived stress were associated with the mediator/moderators of glycemic control (higher self-care, access to care and processes of care).

This study provides the first validation of a conceptual framework for the relationship between socioeconomic and psychological components of social determinants of health and diabetes outcomes. While the mechanisms hypothesized in the model were based on literature, a validation with a sample of 615 patients from two clinics in the southeast US provides further support for the importance of social determinants of health in patients with type 2 diabetes and the need for clinicians to consider these factors during care. Based on these findings, there are direct and indirect pathways through which social determinants influence outcomes. This suggests a need to expand the focus of interventions from mainly lifestyle and self-care based to include access to and processes of care. In addition, these findings suggest interventions should take into account psychosocial factors such as diabetes distress and self-efficacy, which have an independent influence on glycemic control. Psychological interventions have shown effectiveness in both psychological distress and glycemic control in patients with type 2 diabetes [42, 43]. These results suggest clinicians may need to consider multi-component interventions to improve outcomes, incorporating both behavioral and psychological strategies. Addressing social determinants of health, such as social support, which have an indirect effect on glycemic control through their influence on access to and processes of care may be an additional way to impact diabetes outcomes. While the ADA recommends a psychological assessment for patients with diabetes [3], these results suggest the need for a more comprehensive and ongoing assessment of the social determinants influencing patients’ ability to actualize good health outcomes.

While these results correspond to the literature suggesting a relationship between social determinants of health and diabetes outcomes [17–19], they build on the current literature by providing a more comprehensive view of the field. Similar to the results of two path analyses conducted with populations in Turkey, Iran and China, our results indicated the importance of self-efficacy and social support [18, 44, 45]. By expanding the number of variables investigated, we found the importance of these factors on multiple aspects that influence health outcomes, rather than self-care alone. In addition, we validated the presence of a direct pathway between social determinants and health outcomes, including the direct influence of psychological stress on glycemic control. The positive association of diabetes distress and negative association of fatalism suggest a pathway that may be mediated and moderated by various factors. For example, differences in diabetes duration or socioeconomic status may influence the relationship between distress and glycemic control resulting in a positive association when a negative association would have been hypothesized. More work is needed to understand the influences on these relationships in order to develop interventions that appropriately address the social determinants impacting diabetes outcomes.

There are limitations that should be acknowledged. First, the study was conducted in the southeastern United States and should be conducted in different regions and population groups. Second, though the model suggests causal relationships between variables, the data used in this study was cross-sectional and cannot address causality. Non-experimental designs can be analyzed using path analysis, but they do not provide evidence of causation and interpretation of results should be realistic and within the confines of the data [40]. As such, future work should be conducted using longitudinal data.

## Conclusions

In conclusion, this study validated a conceptual framework of the relationship between social determinants of health and diabetes outcomes. Social determinants are contextual factors, which may vary by region and additional work is needed to fully understand these relationships globally. Results from this study suggest future interventions may benefit from including multiple factors including in the model, such as psychological factors and access to care rather than focusing on self-care. Consistent with the model, social determinants of health were directly associated with glycemic control, in addition to being associated with self-care, access to care and processes of care which are themselves associated with glycemic control. Interventions taking these factors into account are more likely to be effective at helping patients with type 2 diabetes achieve good outcomes.
